# The Oscillating Component of the Internal Jugular Vein Flow: The Overlooked Element of Cerebral Circulation

**DOI:** 10.1155/2015/170756

**Published:** 2015-12-09

**Authors:** Francesco Sisini, Eleuterio Toro, Mauro Gambaccini, Paolo Zamboni

**Affiliations:** ^1^Department of Physics and Earth Sciences, University of Ferrara, Via Saragat 1, Ferrara, Italy; ^2^Vascular Diseases Center, University of Ferrara, Via Aldo Moro 8, Cona, 44124 Ferrara, Italy; ^3^Laboratory of Applied Mathematics, DICAM, University of Trento, Via Mesiano 77, 38100 Trento, Italy

## Abstract

The jugular venous pulse (JVP) provides valuable information about cardiac haemodynamics and filling pressures and is an indirect estimate of the central venous pressure (CVP). Recently it has been proven that JVP can be obtained by measuring the cross-sectional area (CSA) of the IJV on each sonogram of an ultrasound B-mode sonogram sequence. It has also been proven that during its pulsation the IJV is distended and hence that the pressure gradient drives the IJV haemodynamics. If this is true, then it will imply the following: (i) the blood velocity in the IJV is a periodic function of the time with period equal to the cardiac period and (ii) the instantaneous blood velocity is given by a time function that can be derived from a flow-dynamics theory that uses the instantaneous pressure gradient as a parameter. The aim of the present study is to confirm the hypothesis that JVP regulates the IJV blood flow and that pressure waves are transmitted from the heart toward the brain through the IJV wall.

## 1. Background

The evaluation of the jugular venous pulse (JVP), defined as the movement of expansion of the jugular vein due to changes in pressure in the right atrium, provides valuable information about cardiac haemodynamics and filling pressures [1], characteristic wave patterns pathognomic of cardiac diseases [2], and an indirect estimate of the central venous pressure (CVP). The JVP evaluation can be useful in the diagnosis and/or prognosis of many heart diseases [3]. Such a pulse consists of three positive waves (*a*, *c*, and *v*) and two descents, defined, respectively, as *x* and *y*. Wave *a* corresponds to the atrial contraction and is synchronized with the *P* wave of the electrocardiogram (ECG). Descent *x* corresponds to the lowering of the atrioventricular septum, interrupted by a small positive wave *c* in relation to the closure of the tricuspid valve; the third wave *v* corresponds to the cardiac systole and is followed by the *y* descent which corresponds to the opening of the tricuspid valve.

In a recent paper [4] we proved that JVP can be obtained by a simple ultrasound (US) B-mode investigation that consists in measuring the cross-sectional area (CSA) of the IJV on each sonogram of video clip acquired in the transversal plane. In that paper, we acquired the time-dependent CSA datasets of three healthy subjects and then calculated the autocorrelation function of the datasets to show that they were periodic and finally we showed that their wave form presented the same *a*, *c*, and *v* waves as the JVP. Our study gave a quantification of the IJV CSA variation during the cardiac cycle. We also have seen that the IJV perimeter, measured on each sonogram of the video clip, was correlating with the IJV CSA (*R* > 0.9). On this point, we believe that it is desirable to have a confirmation of this finding also using a different imaging modality. However, a direct explanation of this finding is that, when in supine position, the pulsation of the IJV is a distension of its wall. This result is very important because it means that there is a time varying transmural pressure whose effect is clearly visible and cannot be neglected; in fact, large changes in transmural pressure are required to induce CSA deformation accompanied by a stretching of the wall [5, 6]. If this is true then the following points are also true: (i) the blood velocity in the IJV is a periodic function of the time with period *T* equal to the cardiac period and (ii) the instantaneous blood velocity is given by a time function that can be derived from a flow-dynamics theory that uses the instantaneous pressure gradient as a parameter, for example, the linear Womersley solution of the Navier-Stokes equations [7].

**Figure 1:**
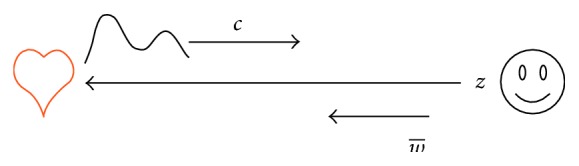
Schematic representation of the heart-head axis. A pressure wave propagating from the heart toward the head with velocity *c* is represented together with the direction of the internal jugular vein blood velocity $\bar{w}$ that propagates from the head toward the heart.

It is worth noting that, in an elastic medium as is the IJV wall, an impulse that gives rise to a wall distension will be propagated following the wave equation of d'Alembert. The time periodic variation of the CSA and hence of pressure measured for the IJV is hence related to the propagation of pressure waves propagating from the heart toward the brain. Thus, in supine position, when the IJV is distended, the blood flow is governed by an oscillating pressure gradient [7] and pressure waves generated by the cardiac revolution are then transmitted from the heart toward the brain (see Figure 1).

The aim of the present study is to verify such a claim by comparing the blood velocity assessed by the current US Doppler method with the blood velocity calculated using the linear Womersley equations. The correspondence between them will confirm the hypothesis that JVP regulates the IJV blood flow and that theoretically pressure waves are transmitted from the heart toward the brain through the IJV wall.

## 2. Methods

### 2.1. Subjects Scanning and Protocol

We have chosen two subjects, labeled as #1, #2 (a 27-year-old female and a 47-year-old male, resp., with no history of cardiovascular, hepatic, gastrointestinal, renal, and cerebral diseases. ECD screening for CCSVI was completely negative [8–10]), out of our database of US scan of the neck by using My-Lab 70 x-vision system (ESAOTE, Genoa, Italy) with a linear array probe 7.5–11 MHz and Vivid-q ultrasound system (GE Medical Systems Ultrasound, Horten, Norway) equipped with a linear probe (L11-812 MHz). They were chosen for this study because they were representative of a small and normal IJV CSA, respectively (with respect to the average diameter value of about 1.3 cm reported in [11]).

The assessment of the jugular CSA was performed using a B-mode scan in the transverse plane of the right IJV at c5/c6 level. Such region corresponds to the segment close to the junction of the IJV with the subclavian vein. For each subject we recorded first a transversal video clip of the CSA (i.e., a sonogram sequence) and immediately after a velocity spectral Doppler trace, both for at least four cardiac cycles. The trace was obtained selecting the “mean velocity mode,” which automatically produces a weighted average of the velocity over the whole US reflected spectrum. This results in a green trace (see Figure 3) that corresponds to the blood velocity averaged across the sample volume. The latter is selected to roughly correspond to the IJV lumen.

This study was conducted in accordance with the Ethical Standards of the Committee on Human Experimentation of the Azienda Ospedaliera Universitaria di Ferrara. All the volunteers signed an informed consent form.

### 2.2. Pulsed Flow-Dynamics Assumptions

Following the original paper of Womersley [7], we denote as $\bar{w}(t)$ the instantaneous blood velocity in direction *z* and averaged over the IJV CSA. The velocity $\bar{w}(t)$ is calculated, at any time *t*, using the Womersley equations, for which the time-dependent pressure gradient ∂*p*(*t*, *z*)/∂*z* is required. The internal pressure *p*(*t*, *z*) was here assumed to be linearly related to the CSA(*t*, *z*) [6, 12, 13] as follows:

(1) $$\begin{array}{c} p\left(\;t,z\;\right)=p_{0}+\frac{1}{C^{\prime }}\times \text{CSA}\left(\;t,z\;\right), \end{array}$$

where *p*_0_ is a convenient additive constant and *C*′ is the IJV compliance for unit of length defined as

(2) $$\begin{array}{c} C^{\prime }=\frac{\partial \text{CSA}}{\partial p}. \end{array}$$

Given a pressure wave velocity *c*, the wave equation states that

(3) $$\begin{array}{c} \frac{\partial p}{\partial z}=\pm \frac{1}{c}\frac{\partial p}{\partial t}. \end{array}$$

Substituting (1/*C*′)CSA(*t*, *z*) from (1) into (3), the pressure gradient ∂*p*/∂*z* is obtained. The minus sign on the right side of (3) represents a wave moving toward the positive direction of the *z*-axis while the plus sign represents a wave moving toward the negative *z*. In order to simplify the interpretation of the results, in this paper we chose to assume the positive direction of the *z* from the head toward the heart (see Figure 1). The pressure waves are generated from the heart pulsation and propagate toward the jugular vein in the negative *z* direction; for this reason they are represented by positive sign in (3). In this work we neglect the effects of reflected waves propagating from the brain toward the heart.

Moreover, the Womersley equations only give the instantaneous oscillating part of the blood velocity. The net instantaneous value of the blood velocity is given by summing the steady velocity with the oscillating velocity. The steady component of the velocity can be obtained by the Poiseuille law once the pressure gradient between the head and the heart is known. In this study we are not interested in a full assessment of the mean velocity so only the oscillating velocity is considered and analyzed.

Having assumed a positive *z* from the head to the heart, the net blood velocity is positive (see Figure 1).

### 2.3. IJV Blood Velocity Assessment: The Oscillating Component

For each subject, the CSA dataset, representing the instantaneous value of the CSA during a cardiac cycle, was derived from the acquired video clip using the semiautomatic algorithm described in [4]. The period *T* of the IJV pulsation was obtained by its discrete Fourier transform calculated using the Grace software [14]. Then we write *τ* = 2*π*(*t*/*T*) − *π* so that *τ* runs from −*π* to *π* during one pulse period. The mean CSA, denoted by $\bar{CSA}$, over a period *T* is calculated. The Fourier coefficients of pressure gradient ∂*p*/∂*z* up to the tenth harmonic are calculated. These coefficients are then converted to modulus *M* and phase *ϕ*. The blood velocity is then calculated from Equation (4) in [7] by substituting the pressure gradient expressed as a Fourier series. The blood velocity $\bar{w}(t)$ is given by the summation of ten terms as in Equation (27) in [7]:

(4) w¯τ,z=−CSA¯τ,z2πM10′μα2∑m=1m=10Mmsin⁡mτ+ϕm+ϵ10,

where we have substituted the term *πR* ^2^ in [7] by CSA(*τ*), which represents the time-dependent vein CSA. The parameter *μ* is the blood viscosity (0.04 Pa), *m* that runs from 1 to 10 is the order of the Fourier coefficient, and *M*_10_′ and *ϵ*_10_ are derived from the Bessel function and tabulated in [7]. Finally, *α* is the well-known Womersley number given by

(5) $$\begin{array}{c} \alpha =R\sqrt{m\frac{2\pi \rho }{T\mu }}, \end{array}$$

where *ρ* is the blood density (1.05 g/mL) and $R=\sqrt{\bar{CSA}/\pi }$.

### 2.4. Spectral Doppler Averaged Velocity

Below we describe the procedure adopted to obtain a dataset sampled from the US Doppler velocity trace. Common commercial US systems do not allow the Doppler dataset to be exported; for this reason, in this work, the mean velocity dataset is obtained by digitally processing the image shot of the Doppler trace. The mean blood velocity $\left(\bar{w}(t)\right)$ is represented as a line overlapping with the spectral Doppler trace (Figure 3). The line is composed of *m* pixels. An in-house developed procedure identifies the pixel belonging to the line by its RGB values. The coordinates *x*_*k*_ and *y*_*k*_ of each pixel were automatically recorded by the procedure, where *k* is an index going from 1 to *m*. The function $\bar{w}(t)$ has been obtained from *x*_*k*_ and *y*_*k*_ values using the following procedure:

(6) tk=xkPS

(7) wk=ykPW×100,

where *k* is the index for the pixels and *PS* is the distance in pixels between two time divisions (separated by 1 s) of the time axes while *PW* is the distance in pixels between the 0 and 100 cm/s divisions, measured along the *y*-axis. Combining the two expressions in (6) produces

(8) $$\begin{array}{c} \bar{w}\left(\;t_{k}\;\right)\equiv {\bar{w}}_{k}=\frac{y_{k}}{PW}\times 100. \end{array}$$

The sampled Doppler velocity dataset $\left\{{\bar{w}}_{k}\right\}$ is then plotted together with the calculated Womersley averaged velocity ($\bar{w}(t)$). The instant of time corresponding to the maximum value of velocity was detected for both the Doppler and $\bar{w}(t)$ and it was used as reference marker to overlap the two plots.

#### 2.4.1. Detailed Calculation

The detailed algorithm of all the calculations needed to produce the following results is reported in the appendix.

## 3. Results

### 3.1. IJV Blood Velocity Assessment

**Figure 2:**
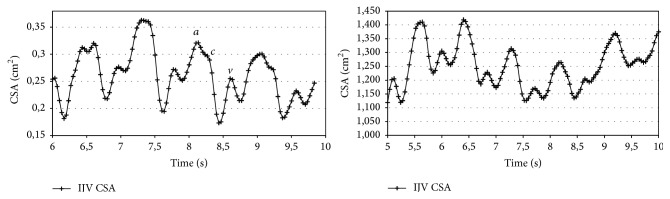
Instantaneous cross-sectional area (CSA) of the internal jugular vein measured using ultrasound B-mode for about 5 seconds.

Each result is produced for both subjects #1 and #2 and presented in the text following such order. Each figure starting from Figure 2 is divided into top and bottom for subjects #1 and #2, respectively. The main numerical results are summarized in Table 1.

The US-JVP are plotted for both subjects in Figure 2; their repetition periods are 0.9 and 0.92 s for subjects #1 and #2, respectively; the time averaged cross-sectional areas $\bar{CSA}$ are 0.25 and 1.25 cm^2^. Both subjects show clearly visible *a* and *v* waves, while the *c* one is barely detectable.

The time derivatives of the internal pressure and pressure gradient were obtained from the CSA dataset as described in the appendix.

The mean blood velocity $\bar{w}$ was calculated following (4) and is plotted together with the CSA in Figure 4. It is worth noting that for both subjects the blood velocity changes inversely with respect to the IJV CSA; that is, when the IJV CSA goes toward its maximum the blood velocity goes toward its minimum and vice versa.

In Figure 4, $\bar{w}$ is plotted together with the pressure gradient ∂*p*/∂*z*.

### 3.2. Spectral Doppler Averaged Velocity

**Figure 3:**
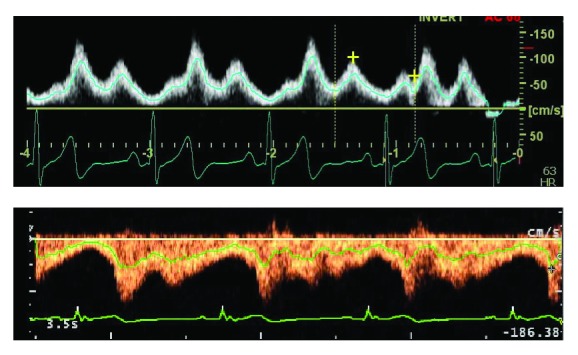
Ultrasound spectral Doppler trace of the right internal jugular vein blood flow. The mean velocity trace is highlighted.

**Figure 4:**
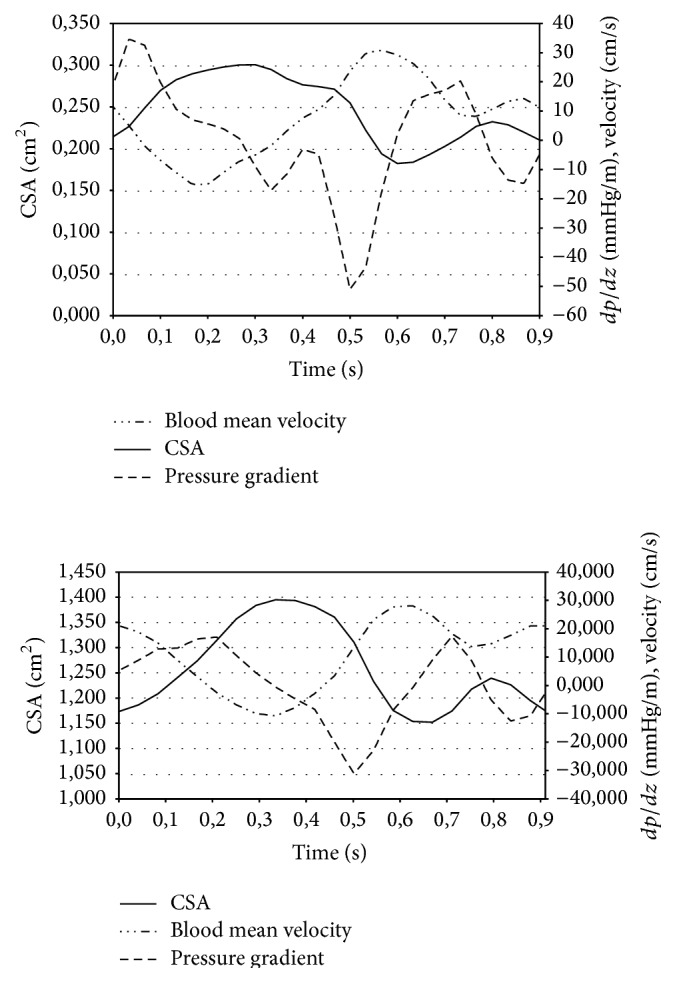
The pressure gradient (*dp*/*dz*) calculated using the “tube-law” is plotted together with the internal jugular vein blood mean velocity ($\bar{w}$) calculated using Womersley equations and the cross-sectional area (CSA). A single cardiac cycle is reported.

Figure 3 depicts the spectral Doppler velocity traces. The time periods of such traces turned out to be 0.95 and 0.88 s for subjects #1 and #2, respectively (see Table 1). Such values are very close to that of the US-JVP period; this is an expected result since the spectral Doppler trace was acquired as soon as the B-mode IJV investigation was completed so as to avoid changes in physiological conditions.

**Figure 5:**
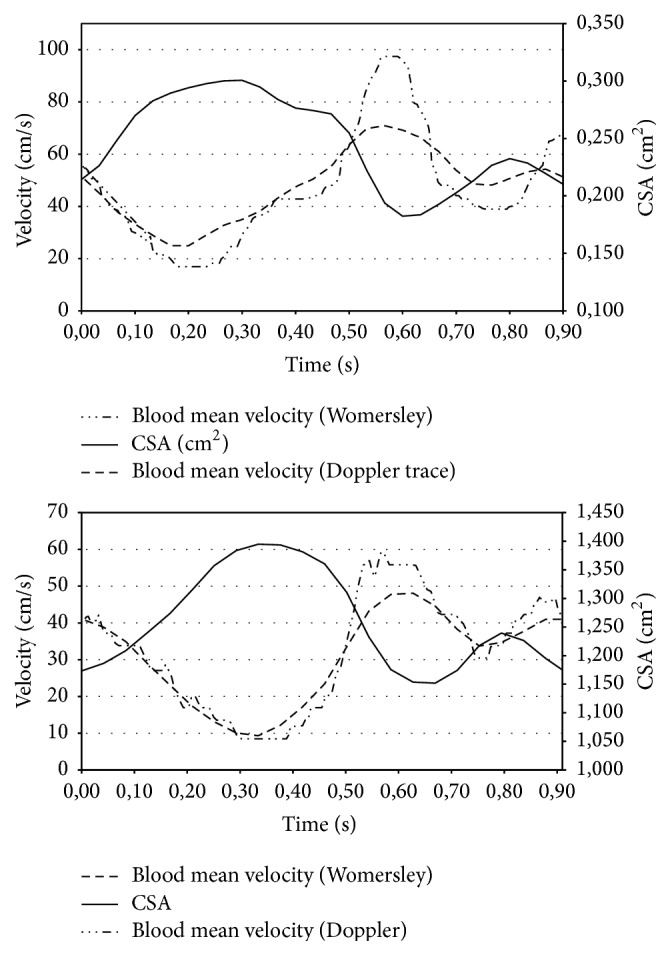
Ultrasound spectral Doppler mean velocity ($\bar{w}$) is plotted together with the internal jugular vein blood mean velocity ($\bar{w}$) calculated using Womersley equations. A single cardiac cycle is reported.

It is evident that the traces of both subjects #1 and #2 show a “two-wave” profile.  Figure 5 depicts the blood velocity sampled from the spectral Doppler versus the calculated ones for subjects #1 and #2, respectively. For both subjects, sampled and calculated velocity values show the same time dependence or, in other words, the same wave form. A reasonable agreement of their amplitudes was obtained by requiring a CVP up to 10 mmHg (see Appendix A.5 for details).

## 4. Discussion

### 4.1. IJV Blood Velocity Assessment

The main result of the present study is the well apparent relationship between the blood velocity in the IJV measured by the current Doppler system and the same parameter derived from its pulse. For both subjects the presence of the two positive peak waves, “*a*” and “*v*” waves, respectively, in the US-JVP (the CSA diagram), generates two descents in the assessed velocity time diagram within the same cardiac period. Although the relationship between the JVP and the variation of pressure in the heart has been known since 1902 [15], until now, this finding has not been taken into consideration for assessing the brain outflow. Currently, the Doppler flow quantification is provided by the product of the time average velocity of flow and the CSA [16–19]. This quantitative approach currently provides an accepted objective criterion for assessing the flow rate. However, despite the pulsating variation during the cardiac cycle of the IJV CSA, the flow, according to this method, is calculated by using just a single CSA value. This raises the question as to whether overlooking the pulsatile variation of the IJV CSA may affect the US evaluation of cerebral venous return in the clinical setting. In addition, the results of the present study further corroborate the role of the mechanical propagation of the cardiac contraction in the upward direction. This wave propagation is not haemodynamically negligible because the present study clearly demonstrates how this phenomenon influences blood flow velocity. There are several studies which suggest that flow waves directed up towards the brain can contribute to neurodegenerative diseases by affecting cerebrospinal fluid absorption and/or brain perfusion, such as Alzheimer [8, 9, 20].

Our data clearly demonstrates how the assumption of the Poiseuille theorem in the case of pulsatile flow may expose the flow assessment to error. We focused our study on US but of course the MRV methodology used for IJV flow assessment could also be affected by the same incorrect assumption. We know that the distal segment of the IJV is characterized by significant CSA variation over time, an effect linked with the transmission of pressure waves from the right atrium. This phenomenon, very well known as JVP, is strongly connected with important regulators of the cardiovascular function: degree of filling, heart pulsation, and capacity of emptying of the IJV.

Moreover, the positive waves “*a*,” “*c*,” and “*v*” clearly show a brain directed propagation of pressure waves with significance never investigated, as yet. We know that propagation is greater when the degree of filling of the venous system is higher and/or when the heart function is compromised. Acute symptoms in the latter rapidly appear in the pulmonary function but we have never investigated if this may have significance for cognitive or neurological decline already described in this particular category of patients. We speculate that cardiac, carotid, and jugular signals should be synchronized and used to assess the circulatory axis between the heart and brain, with significant changes in current clinical practice, in neuroimaging as well.

The results reported here can be reproduced using MRV because CSA and blood velocity can be measured at each instant of time as we did.

### 4.2. Pulsed Flow-Dynamics Assumptions

The Womersley approach presented in [7] derives the blood velocity from the pressure gradient assuming a rigid tube as a model. Such a model accounts for the pressure gradient time dependence but neglects the movement of the tube wall in the longitudinal and lateral direction. In later papers, Womersley made corrections accounting for such variability [13, 21] resulting in a new equation having the same form as the former (see Equation (12) in [13]) and producing a flow about 10% higher in magnitude. In this paper we choose to follow the simpler approach presented in [7] for three reasons: (1) the cited paper is widely known and widely adopted, (2) the presented analytical model can be easily implemented making our results easily reproducible, and (3) such a model results in an easily treatable linear equation and its results also hold for pulsing flow in an elastic tube [13, 21].

However, we encourage the adoption of more sophisticated models when the blood flow has to be calculated for clinical reasons (see, e.g., [12]).

The assumption of simple linear or quadratic relationships between CSA and pressure seems to be correct in the present case. In fact, considering the well-known “tube-law” [6], the CSA/perimeter correlation means that IJV pulsation lies in the curve region (i) where the transmural pressure is greater than the buckling pressure (see Figure 3 in [5]); hence a linear relationship between pressure and CSA is straightforward. On the other hand, the elliptical shape of the IJV during its pulsation could suggest that the transmural pressure is below the buckling value; hence region (ii) could also be affected by the pulsation. Of course this finding needs to be further investigated.

### 4.3. Implication for Pressure Wave

The oscillating nature of the IJV is potentially relevant in the understanding of the relationship between brain drainage and several neurological disorders [22–29]. In particular, the effect of pulsatile flow has been recently hypothesized to be associated with retrograde hypertension transmitted from the IJV which, presumably, underlies transient global amnesia [25] and perhaps other neurological disorders, as recently hypothesized [30–32].

## Figures and Tables

**Table 1:** Time period for jugular venous pulse (JVP) and spectral Doppler (SD) periodic diagram is reported for two healthy subjects together with the time averaged internal jugular vein (IJV) cross-sectional area (CSA) and the wave propagation velocity *c*.

Subject	JVP period (s)	SD period (s)	$\bar{CSA}$ (cm^2^)	*c* (m/s)
1	0.90	0.95	0.25	1.6
2	0.91	0.88	1.25	2.6

## Appendix

### A. Velocity Calculation Detailed Procedure

#### A.1. IJV Elasticity Model

In this paragraph we present an expression for the vein volumetric compliance *C*, that is, the ratio of volume variation Δ*V* with respect to pressure variation Δ*P*, and consequently for the compliance per unit of length *C*′. We assume

(A.1) V=lCSA¯ΔV=lΔCSA

that implies the idea that the vein may change in radius but not in length. From the definition of volumetric compliance *C* it follows that

(A.2) C=ΔVΔpΔp=lΔCSAC.

The coefficient of functional elasticity *E* is defined as

(A.3) $$\begin{array}{c} E\equiv \frac{V\Delta p}{\Delta V}; \end{array}$$

then, using the definitions given in (A.1), we have

(A.4) $$\begin{array}{c} \Delta p=\frac{\Delta \text{CSA}}{\bar{\text{CSA}}}E. \end{array}$$

From the two definitions of Δ*p* given in (A.2) and (A.4) it follows that

(A.5) $$\begin{array}{c} \frac{1}{C^{\prime }}=\frac{E}{\bar{\text{CSA}}}. \end{array}$$

For cylindrical geometry, the relationship between the Young Modulus *M* and *E* can be assumed to be [33]

(A.6) $$\begin{array}{c} M=\frac{2R}{h}E, \end{array}$$

where *h* is the vein wall thickness. The pressure wave velocity *c*, that is, the velocity of propagation of the JVP in the jugular vein, is calculated following the Moens-Korteweg equation:

(A.7) $$\begin{array}{c} c=\sqrt{\frac{M}{\rho }\times \frac{h}{2R}} \end{array}$$

which for the assumed relationship between *M* and *E* is dependent only on the parameters *E* and *ρ*:

(A.8) $$\begin{array}{c} c=\sqrt{\frac{E}{\rho }}. \end{array}$$

The central venous pressure (CVP), measured close to the IJV, is known to vary up to 5 mmHg during the cardiac cycle. Since Δ*p* during a cardiac cycle is given by

(A.9) $$\begin{array}{c} \Delta p=\frac{1}{C^{\prime }}\left(\;\max\left(\;\text{CSA}\left(\;t\;\right)\;\right)-\min\left(\;\text{CSA}\left(\;t\;\right)\;\right)\;\right) \end{array}$$

then

(A.10) $$\begin{array}{c} C^{\prime }=\frac{\max\left(\text{CSA}\left(t\right)\right)-\min\left(\text{CSA}\left(t\right)\right)}{5}. \end{array}$$

Equation (A.10) can also be used with (A.5) to determine *E* which is given as

(A.11) $$\begin{array}{c} E=\frac{\bar{\text{CSA}}}{C^{\prime }}. \end{array}$$

#### A.2. Parameter Data

The following parameter value has been assumed [7, 33]:

(i) Blood viscosity *μ* = 4.5 × 10^−3^ Pa × s.
(ii) Blood density *ρ* = 1.04 × 10^3^ kg/m^3^.
(iii) Vein wall thickness *h* = 0.5 mm.

#### A.3. Input Data

From the US transversal scan of the IJV the following data represents the input for the velocity calculation procedure:

(i) The US-JVP, consisting in a dataset {CSA_*i*_} with *i* running from 1 to *N*, where *N* is the number of sonograms in the US video clip with each element CSA_*i*_ of the dataset representing the IJV area measurement on the *i*th sonogram, in pixels.
(ii) The US video clip duration (Δ*tV*) in seconds.
(iii) The US sonogram pixel dimension (*ps*) in cm.

From the longitudinal US Doppler trace, consider the following:

(i) The space averaged velocity time diagram, consisting in a dataset $\left\{{\bar{w}}_{i}^{\prime }\right\}$ in pixels.
(ii) The distance (*dd*) between two divisions in the time axis of the Doppler trace.

#### A.4. Cross-Sectional Area

(1) {CSA_*i*_} dataset discrete Fourier transform (DFT) is operated (e.g., using Grace software).
(2) The sonogram repetition period (*TN*) of the {CSA_*i*_} dataset is obtained by the maximum of the DFT produced at (1).
(3) The sonogram time interval (Δ*t*) is calculated as Δ*t* = Δ*tV*/*N*.
(4) The {CSA_*i*_} dataset time repetition period is then calculated as *T* = *TN* × Δ*t*.
(5) The {CSA_*i*_} dataset is multiplied by the pixel area (*ps* ^2^) in order for each element CSA_*i*_ of the dataset to represent the IJV area measurement on the *i*th sonogram, in cm^2^.
(6) The differential CSA dataset {ΔCSA_*i*_} is obtained as {ΔCSA_*i*_} = ΔCSA_*i*+1_ − ΔCSA_*i*_.
(7) The discrete partial time derivative {ΔCSA_*i*_/Δ*t*} dataset is obtained dividing the {ΔCSA_*i*_} by Δ*t*.
(8) Average CSA is calculated as $\bar{CSA}=\sum_{i}^{TN}CSA_{i}/TN$.
(9) The average vein diameter is calculated as $d=2\times \sqrt{\bar{CSA}/\pi }$.

#### A.5. Pressure

The following steps focus on the pressure dataset calculation:

(1) The compliance for unit of length is calculated as *C*′ = (Max({CSA_*i*_}) − Min(CSA_*i*_))/CVP_max_, where CVP_max_ is a convenient parameter representing the maximum value reached by the CVP during the cardiac cycle. A typical value is 5 mmHg.
(2) The pulse wave velocity (*c*, lower case) is calculated as $c=\sqrt{M/\rho \times h/d}=\sqrt{E/\rho }$.
(3) Time derivative pressure dataset, in unit of mmHg, is given by {Δ*p*_*i*_/Δ*t*} = (1/*C*′){ΔCSA_*i*_/Δ*t*}.
(4) It is then converted into Pa by multiplying the {Δ*p*_*i*_/Δ*t*} dataset by 133.322 Pa/mmHg.
(5) The pressure gradient along the *z*-axis direction is calculated as {Δ*p*_*i*_/Δ*z*} = ±(1/*c*){Δ*p*_*i*_/Δ*t*}.
(6) The parameter *x* is defined as *x* = 2*π*((*i* − 1) × Δ*t*)/*T* − *π*.
(7) The pressure gradient is then defined as DZP(*x*) = Δ*p*_*i*_/Δ*z*.

#### A.6. Pressure Gradient Fourier Series

The pressure gradient is then decomposed in its Fourier terms as

(A.12) $$\begin{array}{c} \text{DZP}\left(\;x\;\right)=a_{0}+\overset{n}{\underset{m=1}{\sum }}M_{m}\cos\left(\;m\times x+\phi_{m}\;\right). \end{array}$$

The series coefficients are calculated as follows:

(1) *a*_0_ = (2/2*π*)∑_*i*=1_^*TN*^(Δ*p*_*i*_/Δ*z*) × (2*π*/*TN*).
(2) *a*_*m*_ = (2/2*π*)∑_*i*=1_^*TN*^(Δ*p*_*i*_/Δ*z*)cos(*m* × *x*) × (2*π*/*TN*).
(3) *b*_*m*_ = (2/2*π*)∑_*i*=1_^*TN*^(Δ*p*_*i*_/Δ*z*)sin(*m* × *x*) × (2*π*/*TN*).
(4) $M_{m}=\sqrt{a_{m}^{2}+b_{m}^{2}}$.
(5) *ϕ*_*m*_ = −arctg(*b*_*m*_/*a*_*m*_).

#### A.7. Blood Velocity Fourier Series

The blood velocity expression is obtained by the pressure gradient expression as

(A.13) w¯x=−CSA¯π·1μa0+∑m=1nMmM10′α2sin⁡mx+ϕm+ϵ10.

(1) The parameter *α* for each harmonic *m* is calculated as $\alpha_{m}=(d/2)\sqrt{m(2\pi /T)(\rho /\mu )}$.
(2) The coefficients *M*_10_′/*α* ^2^ and *ϵ*_10_ are tabulated in [7] for each *α* value from 0 to 10. For *α* > 10 the asymptotic expression was used [7].

## Abbreviations

### Acronyms

CCSVI: Chronic venous cerebrospinal insufficiency
CSA: Cross-sectional area
CVP: Central venous pressure
DFT: Discrete Fourier transform
DZP: Pressure gradient
ECD: Echo color Doppler
ECG: Electrocardiogram
IJV: Internal jugular vein
JVP: Jugular venous pulse
MRV: Magnetic resonance venography
*PS*: Distance in pixels between two time divisions
*PW*: Distance in pixels between the 0 and 100 cm/s divisions
*R*: Pearson coefficient
RGB: Red Green Blue
*TN*: Sonogram repetition period
US: Ultrasound.

### Symbols

*α*: Womersley number
*c*: Pressure waves velocity
*C*: Compliance
*C*′: Compliance for unit of length
Δ*t*: Sonogram time interval
*E*: Coefficient of functional elasticity
*h*: Vein wall thickness
*μ*: Blood viscosity
*M*: Young Modulus
*ps*: Pixel dimension
*p*: Pressure
*ρ*: Blood density
*T*: Cardiac period
*v*: Volume
$\bar{w}$: Blood flow velocity averaged over the IJV CSA.
